# Integrated single‐cell transcriptomics reveals the hypoxia‐induced inflammation‐cancer transformation in NASH‐derived hepatocellular carcinoma

**DOI:** 10.1111/cpr.13576

**Published:** 2023-11-22

**Authors:** Yuan Liang, Rui Zhang, Siddhartha Biswas, Qingfa Bu, Zibo Xu, Lei Qiao, Yan Zhou, Jiaqi Tang, Jinren Zhou, Haoming Zhou, Ling Lu

**Affiliations:** ^1^ Hepatobiliary Center, The First Affiliated Hospital of Nanjing Medical University, Research Unit of Liver Transplantation and Transplant Immunology, Chinese Academy of Medical Sciences Nanjing Medical University Nanjing China; ^2^ School of Biological Science & Medical Engineering Southeast University Nanjing China; ^3^ Department of Bioinformatics Nanjing Medical University Nanjing China; ^4^ Department of Pancreatic Surgery, Nanjing Drum Tower Hospital The Affiliated Hospital of Nanjing University Medical School Nanjing China; ^5^ Affiliated Hospital of Xuzhou Medical University Xuzhou China

## Abstract

Non‐alcoholic fatty liver disease (NAFLD) has emerged as the primary risk factor for hepatocellular carcinoma (HCC), owing to improved vaccination rates of Hepatitis B and the increasing prevalence of metabolic syndrome related to obesity. Although the importance of innate and adaptive immune cells has been emphasized, the malignant transformation of hepatocytes and their intricate cellular network with the immune system remain unclear. The study incorporated four single‐cell transcriptomic datasets of liver tissues covering healthy and NAFLD‐related disease status. To identify the subsets and functions of hepatocytes and macrophages, we employed differential composition analysis, functional enrichment analysis, pseudotime analysis, and scenic analysis. Furthermore, an experimental mouse model for the transformation of nonalcoholic steatohepatitis into hepatocellular carcinoma was established for validation purposes. We defined CYP7A1^+^ hepatocytes enriched in precancerous lesions as ‘Transitional Cells’ in the progression from NAFLD to HCC. CYP7A1^+^ hepatocytes upregulated genes associated with stress response, inflammation and cancer‐associated pathways and downregulated the normal hepatocyte signature. We observed that hypoxia activation accompanied the entire process of inflammation‐cancer transformation. Hepatocyte‐derived HIF1A was gradually activated during the progression of NAFLD disease to adapt to the hypoxic microenvironment and hepatocytes under hypoxic environment led to changes in the metabolism, proliferation and angiogenesis, promoting the occurrence of tumours. Meanwhile, hypoxia induced the polarization of RACK1^+^ macrophages that enriched in the liver tissues of NASH towards immunosuppressed TREM2^+^ macrophages. Moreover, immunosuppressive TREM2^+^ macrophages were recruited by tumour cells through the CCL15‐CCR1 axis to enhance immunosuppressive microenvironment and promote NAFLD‐related HCC progression. The study provides a deep understanding of the development mechanism of NAFLD‐related HCC and offers theoretical support and experimental basis for biological targets, drug research, and clinical application.

## Graphical abstract

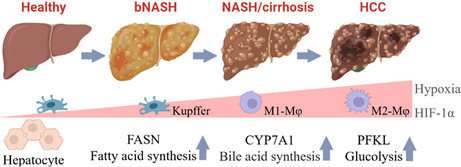

## 1 BACKGROUND

Primary liver cancer ranks as the sixth most common cancer in the world and is the third leading cause of cancer‐related mortality.^1^ Hepatocellular carcinoma (HCC) constitutes the primary histologic subtype of primary liver cancer, accounting for approximately 85%–90% of cases and exhibiting remarkable heterogeneity and invasiveness.^2^ The occurrence of HCC is linked to various risk factors such as persistent infections of hepatitis B and C viruses, alcohol abuse, exposure to dietary poisons, and non‐alcoholic fatty liver disease (NAFLD), among others.^1^, ^3^ With obesity and metabolic disorders becoming increasingly prevalent worldwide, NAFLD has now emerged as the most widespread chronic liver ailment. In contrast to other causes of HCC, NAFLD‐associated HCC primarily affects elderly individuals, often leading to a delayed diagnosis and poor survival prospects.^4^ Notably, the prevalence of NAFLD in China doubled between 2010 and 2020, escalating from 18% to 29%.

NAFLD is a progressive liver disease caused by excessive accumulation of lipids in the liver. Non‐Alcoholic steatohepatitis (NASH) is an inflammatory subtype of NAFLD associated with significant hepatocyte damage and lobular inflammation, with a global prevalence of 1.5%–6.5%. NAFLD‐related HCC has a clear pathogenesis, namely, NAFLD‐NASH‐Fibrosis‐HCC. Xuelian Xiong et al. established an AMLN (amylin liver NASH) diet‐fed mouse disease model and utilized single‐cell sequencing to identify the enrichment of NASH‐associated macrophages (NAMs) in the liver tissue of NASH mice which was closely related to the severity of the disease.^5^ In a NASH mouse model based on a high‐fat and high‐fructose diet, Zhenyang Shen et al. found that Egrl^+^/Ly6a^+^ endothelial cells accumulated and activated hepatic stellate cells during NASH progression.^6^ Shuang Wang et al. discovered that inhibiting the NTRK3‐NTF3 signalling pathway could inhibit the activation of hepatic stellate cells and reverse NASH fibrosis, suggesting that the autocrine signalling factor NTRK3 was an anti‐fibrosis drug target for NASH.^7^ These studies used single‐cell sequencing technology to compare the differences between liver tissues in a disease state of NAFLD and healthy liver tissues, analysing the influence of specific enriched cell subtypes in the NAFLD disease, which provides an important foundation for exploring the pathogenesis and development of NAFLD. However, these studies only focus on a specific stage in disease progression of NAFLD, ignoring the continuity of the development of NAFLD and lacking a complete systematic exploration of the NAFLD disease spectrum. Systematic study of dynamic remodelling of hepatocytes and immune cells in the NAFLD disease spectrum is crucial for comprehending the development of NAFLD‐related diseases, and helping identify the progression from fatty liver to NASH, liver fibrosis, and hepatocellular carcinoma. Furthermore, it could aid in comprehending the mechanisms underlying the development of NAFLD. Examining the changes in the cell composition and the molecular mechanisms in the pathological microenvironment during the development of NAFLD‐related HCC could help identify appropriate therapeutic targets and customize personalized treatment regimens for patients with NAFLD‐related diseases.

## 2 METHODS

### 2.1 Data collection and arrangement

Publicly available single‐cell sequencing datasets were acquired from GSE115469,^8^ GSE159977,^9^ GSE136103,^10^ and GSE156625^11^ which were downloaded from the Gene Expression Omnibus database (GEO, https://www.ncbi.nlm.nih.gov/geo/) for the discovery set. We also collected single‐cell sequencing data contained hepatocytes of mice which fed a high‐fat diet for 17 weeks from GSE182365.^12^ The human RNA expression microarray datasets of NAFLD‐associated diseases GSE89632,^13^ GSE48452,^14^ GSE66676,^15^ and GSE49541^16^ were achieved from the GEO database for validation. The microarray expression dataset of the liver of the NAFLD‐related HCC mouse model was achieved from GSE83596 for validation. The R package AnnoProbe (v0.1.7) was used to convert the probes into gene symbols for subsequent analysis. We gathered the spatial transcriptomics data of two western diet‐fed NAFLD mouse livers and two healthy mouse livers from GSE192742^17^ for validation. Details of the datasets in this study are provided in Table S1.

### 2.2 Single‐cell data preprocessing and quality control

The software package CellRanger (v3.1.0)^18^ was applied to compare the fastq file to the reference genome hg38, complete the quantification of cells and genes and obtain downstream analysis files. The R package Seurat (v4.1.0)^19^ was applied to process the above single‐cell sequencing data. Data processing includes data reading, data quality control, gene and cell filtering, data normalization, identification of highly variable genes, data scaling, principal component analysis (PCA), and Uniform Manifold Approximation and Projection (UMAP) for dimension reduction. First, we use the CreateSeuratObject function to construct a Seurat object, and use this function to remove cells of poor quality, in which the retained genes are expressed in at least 20 cells, and the retained cells detect at least 1000 genes; subsequently, according to the characteristics of the sample, we remove low‐quality cells by the number of genes detected in the sample, the proportion of mitochondria and the total number of molecules detected; then we use the ‘LogNormlize’ method of the NormalizeData function to normalize the data, and the scaling factor is 10,000; then we use the function FindVaribleFeatures to identify 4000 highly variable genes for subsequent analysis; the RunPCA function is used for data dimensionality reduction, and the first 50 PCs are selected for downstream analysis. Finally, the UMAP algorithm is used to display the dimensionality reduction of cells.

### 2.3 Integration of single‐cell transcriptome sequencing data based on the Harmony algorithm

Considering that this study uses multiple publicly available single‐cell transcriptome datasets with significant batch effects, batch correction and dimensionality reduction analysis of the data was performed using the R package Harmony (v0.1.0)^20^ to avoid the impact of batch effects. The Harmony method is an integration algorithm based on iterative clustering, which is suitable for the integration of multimodal single‐cell data from different experimental conditions, different research backgrounds, and different sample sources. Compared with other algorithms, the Harmony algorithm has the advantages of small memory consumption, fast running speed, high sensitivity to rare cells, and embedded analysis.

### 2.4 Pathway enrichment score

The R package Vision (v3.0.0)^21^ was used to evaluate the activation degree of the specified pathway in a single cell, and the signature genes sets of all pathways were downloaded from the Molecular Signatures Database (MSigDB, https://www.gsea-msigdb.org/gsea/msigdb).

### 2.5 Identifying cell types

A collection of classical cell‐specific marker genes was compiled to identify cell types. The classic molecular markers corresponding to each cell type in this study are shown in Table S2. We also referred cluster‐specific differential expressed genes identified by Seurat's ‘FindAllMarkers’ function using the Wilcoxon Rank Sum test that were known to be cell markers to help verify the cell type.

### 2.6 Principal component analysis

Average gene expression levels were calculated for each cluster of hepatocytes, and the R package FactoMineR (v2.4) was used to implement principal component analysis based on the average gene expression value, and the fviz_pca_ind function was used for visualization.

### 2.7 Clusters correlation analysis

R package genesorteR (v0.4.3)^22^ was used to definite the high variable gene of hepatocytes, and calculate the correlation between different hepatocyte clusters with default parameters. The plotCorrelationHeat function was used for visualization.

### 2.8 Identify tumour cells

R package Copykat (v1.0.8)^23^ was applied to distinguish normal hepatocytes from malignant tumour cells. It is an integrated Bayesian segmentation algorithm that can estimate high throughput scRNA‐seq genomic copy number profiles, identifies major clonal subpopulations, and yields normal cell and tumour cell prediction results.

### 2.9 Cell interaction analysis

NicheNet (v1.1.0)^24^ was applied to explore cell–cell interactions. The algorithm collects transcription factors and their receptor target information in public databases including KEGG, ENCODE, PhoshoSite, etc., and predicts cellular interactions by calculating differentially expressed and highly active ligand‐receptor pairs under different conditions. In this study, hepatocytes or tumour cells were defined as the cell types that send signals, and endothelial cells are designated as the cell types that receive signals.

### 2.10 Differential expression analysis and functional annotation

This study used the FindMarkers function in the R package Seurat (v4.1.1) to identify the signature genes of a specified cell population, where a differential gene was defined as a gene with a log fold change greater than 0.25 and expressed in more than 25% of cells compared to the remaining cells. In order to analyse the possible functions of differential expressed genes, EnrichR (https://maayanlab.cloud/Enrichr/)^25^ was applied to annotate the functions. GSEA functional enrichment of transition state cells was analysed using the gseGO function in the R package clusterProfiler (v4.2.2),^26^ in which the signalling pathways with NES > 0 and a significance *p* value < 0.05 after FDR correction were defined as activated pathways and inflammation‐related pathways were visualized using the R package GseaVis (v0.0.1).

### 2.11 Pseudotime analysis and functional annotation

Pseudotime analysis of designated cell subpopulations using Monocle (v2.14.0).^27^ Calculate the highly variable genes and use the ‘DDRTree’ method to reduce the dimensionality of the dataset, fit the optimal cell differentiation trajectory curve, and use the heatmap to visualize the expression distribution and clustering of the highly variable genes in the differentiation trajectory. The R package destiny (v3.8.1) was also used to infer the pseudotime trajectory of hepatocytes. We downloaded the Hallmark pathways gene sets from the MsigDB (http://www.gsea-msigdb.org/gsea/msigdb/) database to analyse the activation of pathways along inferred pseudotime.

### 2.12 TRRUST predicts key transcription factors 

The database TRRUST contains 800 human Transcription factors (TF) and 8444 and 6552 TFs of 828 mouse TFs and the regulatory relationships of their target genes, which can be used to deduce potential regulatory roles between TFs and target genes in humans and mice. We used the TRRUST (https://www.grnpedia.org/trrust/)^28^ to predict the key transcription factors that affect the hepatocytes' malignant transformation.

### 2.13 Single‐cell gene regulatory network analysis

The regulon activity scores of single cells were calculated from the raw count matrix using pySCENIC (v0.9.19)^29^ to predict the activation of cell subpopulation‐specific key regulons. Prediction of protein activation in hepatocytes using regulatory information from the R package DoRothEA (v1.2.2).^30^ Signed transcription factor‐target gene interactions were assigned confidence levels from A to E, representing high confidence to computer prediction support only, and interaction pairs with confidence levels A/B/C were selected for downstream analysis. Regulatory patterns between transcription factors and target genes are demonstrated by the netplot R package (v0.1.1).

### 2.14 Metabolic analysis

scMetabolism (v0.2.1) is an R package suitable for quantification of cellular metabolic activity at single‐cell resolution.^31^ scMetabolism was applied to analyse the activation of KEGG metabolic pathways in hepatocytes. The metabolic pathway activity of each cluster of hepatocytes was represented by the average activity score of metabolic pathways.

### 2.15 Construction of NAFLD‐related HCC model

2‐week‐old C57BL/6 male mice were administrated intricately with Diethylnitrosamine (DEN) at a dose of 25 mg/kg. 4 weeks later, mice were fed a high fat diet (HFD), and sacrificed after 8, 24, and 32 weeks of HFD feeding, respectively. The liver tissues of mice were embedded in paraffin and sliced for HE staining. The results of HE staining were determined by the pathologist for NAFL, NASH, and HCC.

### 2.16 Western blot assay

Proteins were extracted from liver tissues with ice‐cold lysis buffer (50 mM Tris, 150 mM Nacl, 1% sodium deoxycholate, 0.1% sodium dodecyl sulphate, and 1% Triton‐100). The proteins were separated by SDS‐PAGE on 8%–12% polyacrylamide gels and subsequently electrically transferred to a PVDF membrane. After blocking with 5% (w/v) BSA in TBST at room temperature for 1 h, the membranes were then incubated with an appropriate specific primary antibody (anti‐Cyp7a1, Santa Cruz, Cat# sc‐518007; anti Hif1a, Abcam, Cat# ab307829; anti β‐actin, Beyotime, Cat# AA128) at 4°C overnight, followed by incubation with an HRP‐conjugated secondary antibody (anti‐rabbit IgG, CST, Cat #7074; anti‐mouse IgG, CST, Cat# 7076). Detection was performed using an enhanced chemiluminescence kit.

### 2.17 Statistical analysis

All statistical analyses were calculated using R (http://www.r-project.org, v4.1.0). The Wilcoxon rank sum test was used to compare the differences between the two groups. Bonferroni was used to calibrate the multiple hypothesis test, and a *p* value less than 0.05 was considered statistically significant. The Spearman correlation coefficient was used to characterize the correlation, and a *p* value less than 0.05 was considered statistically significant. Asterisks are used to indicate statistical significance (*p < 0.05; **p < 0.01; ***p < 0.001); ns, there was no statistical significance (*p* > 0.05).

## 3 RESULTS

### 3.1 Single‐cell transcriptional atlas of disease progression in non‐alcoholic fatty liver disease‐associated hepatocellular carcinoma

**FIGURE 1:**
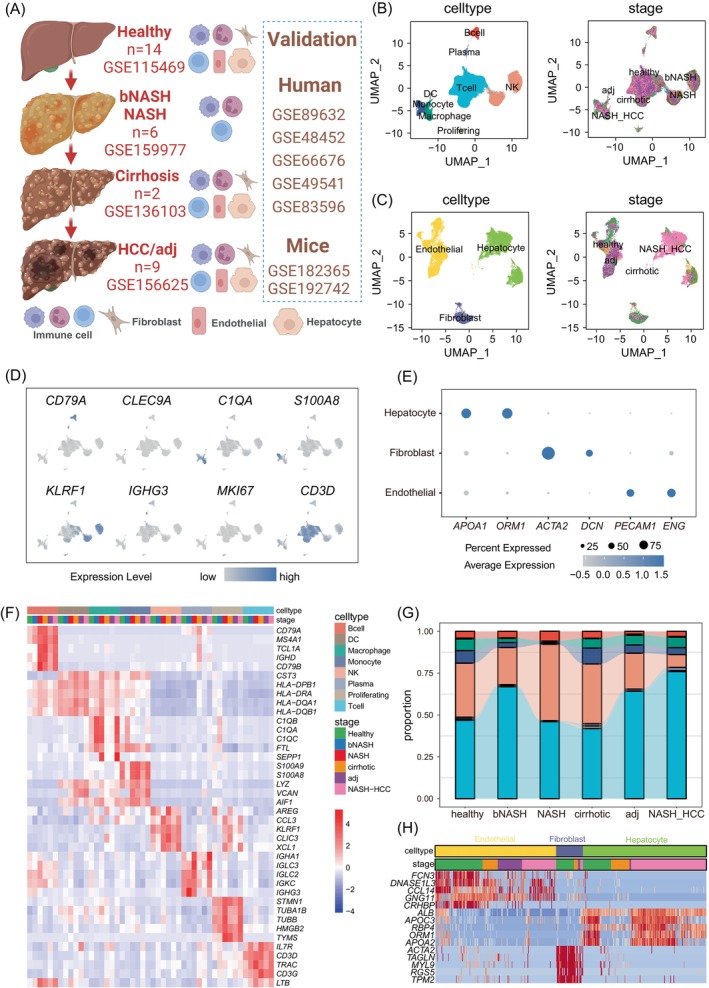
scRNA‐seq analysis of liver samples at different stages of NAFLD‐related HCC development. (A) Schematic representation of data collection. (B) UMAP plot showing the distribution of immune cell types and their disease stages at different stages of NAFLD‐related HCC and different colours represent different cell types or disease stages. (C) UMAP plot showing the distribution of non‐immune cell types and their disease stages at each stage of NAFLD‐related HCC and different colours represent different cell types or disease stages. (D) UMAP plots showing the expression of classical molecular markers of non‐immune cells. (E) The bubble plot shows the classical molecular markers identified by cells, where the size of the point represents the percentage of marker gene expression; the colour depth represents the average expression value of marker gene expression. (F) The heatmap shows the average differential gene expression of immune cells in each group, and the expression values are normalized. (G) Sankey plot showing changes in the proportion of immune cells at different stages of disease progression. (H) Heatmap showing the mean expression of differential genes in non‐immune cells at each disease stage, and the expression values are normalized.

To investigate the pathogenesis of NAFLD‐related HCC, we collected four scRNA‐seq datasets from healthy individuals, boderline NASH (bNASH) patients, NASH patients, NASH‐associated cirrhosis patients and NAFLD‐related HCC patients, which covered the entire process of the development of NASH‐associated hepatocellular carcinoma (Figure 1A). After rigorous quality control, we obtained the transcriptome data of 156,338 cells. Considering that samples from bNASH patients and NASH patients were dissociated and sorted for CD45^+^ cells for scRNA‐seq (Figure 1A), we divided all cells into CD45^+^ immune cells and CD45^−^ non‐immune cells to mitigate potential batch effects of different sequencing strategies on the results (Figure S1A,B). About 80,000 immune cells were divided into 19 clusters after removing batch effects by Harmony and unsupervised clustering (Figure S1C,D). We identified eight major cell types of immune cells by using classical lineage‐specific markers, these cell types included B cells (MZB1; CD79A), plasma cells (IGHG3; IGKC), T cells (CD3E; CD3D; CD3G), NK cells (GNLY; NKG7), macrophages (C1QA; C1QC), monocytes (S100A8; S100A9), dendritic cells (CLEC9A) and proliferating cells (Figure 1B,D). Next, we calculated the differential genes between immune cell subpopulations (Table S3). The results revealed that B cells exhibited high expression of genes like MS4A1 and CD79B, while T cells exhibited high expression of genes such as IL7R and CD3D (Figure 1F). These findings further confirmed the accuracy of cell identification.^32^, ^33^ Comparing the infiltration of immune cells at different stages of NAFLD‐related HCC, we observed significant differences in the composition of immune cells at different stages. Specifically, we observed an enrichment of NK cells in the NASH stage and a decrease in the HCC stage. On the other hand, myeloid cells (macrophages and monocytes) decreased in the NASH stage but significantly increased in the late‐stage liver cirrhosis and HCC stage. These findings are consistent with previous observations^34^, ^35^, ^36^ and suggest that dynamic changes in immune cells play a significant role in the progression of NAFLD‐associated disease (Figure 1G). Additionally, we obtained 23,601 non‐immune CD45^−^ cells, which classified into 13 clusters (Figure S1E,F). Based on classical cell molecular markers reported in the literature^8^ (Figure 1D), these cells were annotated into three cell types: hepatocytes (APOA1; ORM1), fibroblasts (ACTA2; DCN), and endothelial cells (PECAM1; ENG) (Figure 1C,E). Importantly, hepatocytes derived from tumour samples consistently showed high expression of malignant genes KRT7 and PCNA. This observation suggests the malignant transformation of hepatocytes at the transcriptome level towards the end of the disease progression (Figure 1H).

### 3.2 Identification of transitional cells in the progression from NAFLD to HCC

**FIGURE 2:**
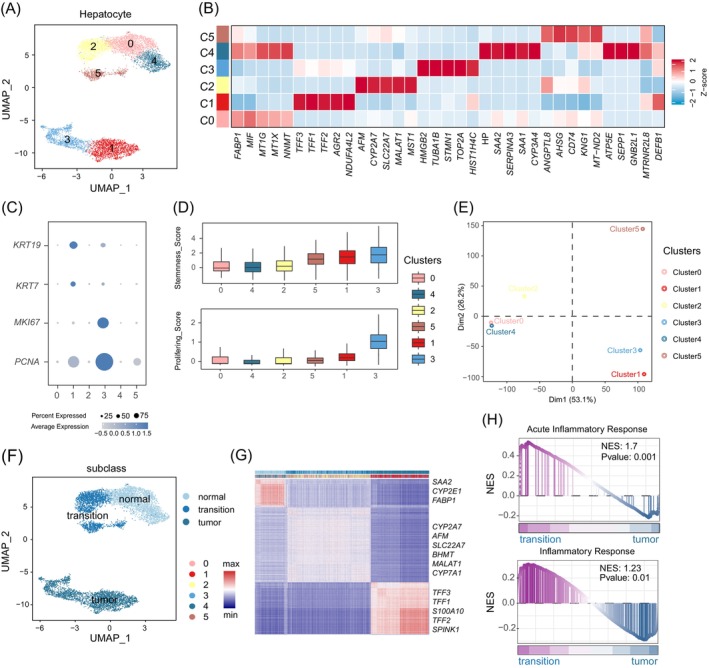
Re‐clustering analysis of hepatocytes. (A) UMAP plot showing the clustering of hepatocytes into six clusters. (B) The heatmap shows the average expression of differential genes in hepatocytes and the expression values were normalized. (C) The bubble plot shows the expression of malignant cell markers in hepatocytes and the colour depth represents the average expression value and the size of the bubble represents the significance. (D) The boxplot shows the distribution of Stemnness_Score and Prolifering_Score of each cluster of hepatocytes. (E) Scatter plot showing the heterogeneity of hepatocytes in each cluster. (F) UMAP plot showing the classification of hepatocytes, with different colours representing different types. (G) Correlation heatmap demonstrating the similarity between hepatocytes. (H) GSEA analysis of the enrichment of Acute Inflammatory Response (GO:0002526) and Inflammatory Response (GO:0006954) gene sets between transitional hepatocytes and tumour cells.

Hepatocytes play a crucial role in maintaining liver homeostasis.^37^ To investigate the heterogeneity of hepatocytes and the potential changes at the transcriptome level during the progression from NAFLD to HCC, hepatocytes were re‐clustered and divided into six subpopulations (Figure 2A). Cluster 0 (C0) and cluster 4 (C4) were present in both normal and tumour samples, while the remaining clusters were primarily derived from tumour samples (Figure S2A,B). Differential expression analysis revealed that both C0 and C4 showed high expression of the endogenous cytoprotectant FABP1, a molecule previously shown to minimize oxidative damage and liver injury in hepatocytes.^38^ In contrast, C1 and C3 exhibited overexpression of trefoil factor family genes (TFF1, TFF2, and TFF3), which are associated with angiogenesis, apoptosis, and tumour progression.^39^, ^40^ Additionally, C1 and C3 showed higher expression of keratin KRT19 and KRT7, as well as proliferation‐related proteins MKI67 and PCNA, suggesting their potential malignancy. C2 highly expressed metabolism‐related genes CYP2A7 and MALAT1 (Figure 2B and Figure S2J), while C3 highly expressed proliferation‐related genes such as STMN1 and TOP2A (Figure 2B,C). To further identify tumour cells, we utilized Copykat to predict copy number variations in hepatocytes and observed that most hepatocytes in C1, C3, and C5 were identified as malignant cells (Figure S2C). Furthermore, we evaluated the stemness and proliferation scores, calculated using the Vision algorithm^41^ based on stemness and proliferation signatures, which revealed higher scores for C1 and C2 hepatocytes (Figure 2D). Based on these findings, we classified C1, C3, and C5 hepatocytes as malignant cells (Figure S2D). Interestingly, C5 hepatocytes exhibited relatively high stemness and proliferation characteristics, as well as copy number variations, while also showing high expression of normal hepatocyte‐related genes such as FABP1 and APOA1 (Figure S2E,F). Subsequently, we explored the correlation between the six hepatocyte subtypes. Principal component analysis (PCA) based on the average expression levels of genes in each hepatocyte cluster indicated that C1 and C3 exhibited relatively similar transcriptome characteristics, as did C0 and C4. On the other hand, C2 and C5 appeared relatively independent (Figure 2E). The correlation analysis also confirmed that C2 and C5 had more similar transcriptome characteristics (Figure S2G). By considering the gene expression profiles of each cluster and the staged characteristics of NAFLD‐related HCC pathogenesis, we designated C0 and C4 as normal hepatocytes, C2 and C5 as transitional hepatocytes representing an intermediate state of malignant transformation, and C1 and C3 as malignant cells (Figure 2F).

To further validate the newly defined transitional cells, we conducted unsupervised clustering of all hepatocytes based on the top 50 differentially expressed genes in each cluster. The result revealed three types of hepatocytes: normal type, transitional type, and malignant type (Figure 2F). Additionally, we calculated the normal hepatocyte signature from the Liver Single Cell Atlas database^42^ and observed that normal hepatocytes had the highest normal hepatocyte score, tumour cells had the lowest score, and transitional cells fell in between. This finding indicated that transitional cells were in an intermediate state of disease progression (Figure S2H). Understanding the characteristics of transitional cells in‐depth is therefore crucial for comprehending the development of NAFLD‐related HCC. GSEA analysis demonstrated that transitional cells activated inflammation‐related pathways, such as acute inflammatory response and inflammatory response compared with tumour cells, and transitional cells downregulated negative regulation of inflammatory response compared with normal hepatocytes (Figure 2H and Figure S2K). This finding suggested that transitional cells were in a state of inflammatory activation. Moreover, function enrichment analysis revealed that transitional cells specifically activated signalling pathways including NOD‐like receptor signalling, PI3K‐AKT signalling, mTOR signalling, Hedgehog signalling, hepatocellular carcinoma, and non‐alcoholic fatty liver disease pathways (Figure S2I). These observations indicated that transitional cells exhibited inflammatory and protumor‐associated features, suggesting their potential transformation into malignant cells.

### 3.3 Transitional hepatocytes activate the bile acid synthesis pathway by regulating CYP7A1

**FIGURE 3:**
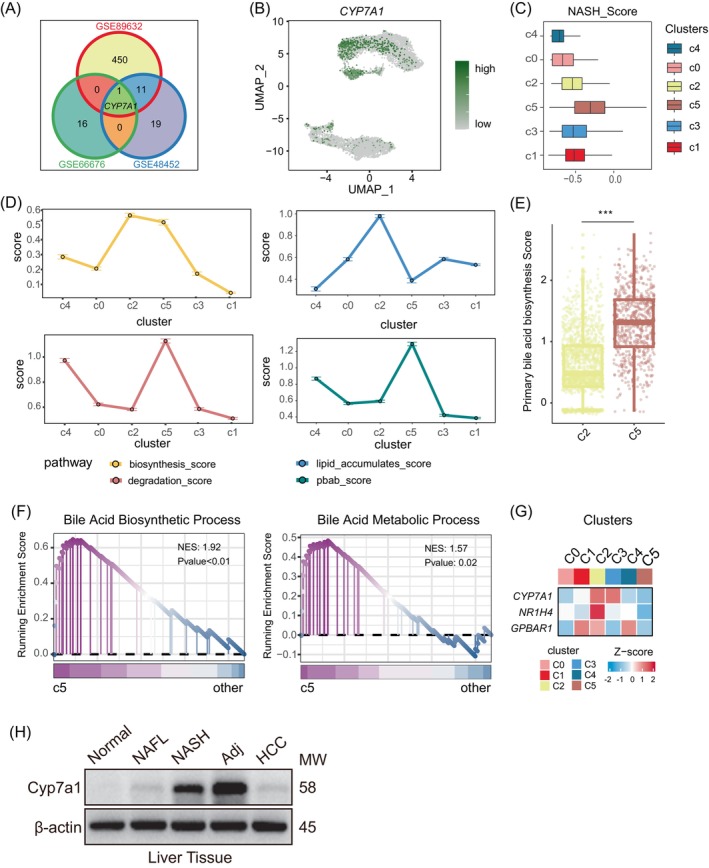
Metabolic analysis of hepatocytes. (A) Venn diagram showing the intersection of differential genes between NASH samples and Normal samples in GSE48452, GSE66676 and GSE89632 datasets. (B) UMAP plot showing the expression of gene *CYP7A1* in hepatocytes. (C) Boxplot showing NASH_Score for each cluster of hepatocytes. (D) The line graph shows the activation degree of fatty acid synthesis pathway, fatty acid degradation pathway, fatty acid accumulation degree and primary bile acid synthesis pathway of each cluster of hepatocytes. (E) Boxplot showing the distribution of primary bile acid synthesis pathway scores of hepatocytes in clusters 2 and 5, with each point representing a cell. (F) GSEA analysis of the enrichment of Bile Acid Biosynthetic Process (GO:0006699) and Bile Acid Metabolic Process (GO:0008206) gene sets between cluster 5 hepatocytes and other hepatocytes. (G) The heatmap shows the average expression of genes which related to bile acid metabolism in hepatocytes and the expression values were normalized. (H) The expression of Cyp7a1 protein in normal liver tissue, NAFL liver tissue, NASH liver tissue, NASH‐HCC‐Adj liver tissue and NASH‐HCC liver tissue were analysed by Western blot, and β‐actin was used as control. *N* = 3/group.

To explore the transcriptome characteristics of transitional cells, we analysed three GEO datasets containing samples from individuals with NASH and healthy livers. By intersecting the results from these differential expression analyses, we identified a single common differential gene called CYP7A1 (Figure 3A, Figure S3A–C). Further examination of CYP7A1 expression in hepatocytes revealed its high expression only in the transition cells (Figure 3B). Furthermore, we utilized the Vision algorithm to analyse the differential gene sets and found that hepatocytes derived from the fifth cluster exhibited significantly higher NASH scores compared to the others (Figure 3C). This suggests that hepatocytes derived from cluster 5 share transcriptome characteristics with hepatocytes in the NASH stage.

CYP7A1 has been recognized as the rate‐limiting enzyme in the bile acid synthesis pathway,^43^ supporting the notion of over‐activation of bile acid synthesis in transitional cells. To investigate the specificity of bile acid synthesis pathway activation, we performed KEGG functional enrichment analysis on each cluster of cells. The results showed that the primary bile acid synthesis, bile secretion, and other related pathways were specifically activated in clusters 2 and 5. In contrast, clusters 1 and 3 activated the p53 signalling pathway, HIF1A signalling pathway, and other tumour‐related pathways, aligning with our previous analysis (Figure S3D). Notably, clusters 1 and 3 exhibited specific activation of the fatty acid degradation pathway, while clusters 2 and 5 specifically activated the fatty acid synthesis pathway, indicating potential changes in fatty acid metabolism in hepatocytes (Figure S3D).

To assess the activation levels of the fatty acid synthesis and fatty acid degradation pathways in hepatocytes, we utilized the Vision algorithm and combined the fatty acid synthesis score and fatty acid degradation score to quantify fat accumulation. The results demonstrated that cells in the second cluster had higher fatty acid synthesis ability, but their fatty acid oxidation levels decreased, indicating excessive fatty acid accumulation in hepatocytes from the second cluster. Conversely, cluster 5 cells exhibited an active fatty acid synthesis pathway coupled with an active fatty acid degradation pathway, effectively avoiding excessive lipid accumulation (Figure 3D). Furthermore, the comparison of fatty acid synthetase (FASN) and CYP7A1 expression in hepatocytes of various clusters provided preliminary verification of the previous results (Figure S3E).

Functional enrichment analysis also indicated highly activated bile acid metabolism‐related pathways in CYP7A1^+^ transitional cells from cluster 5 (Figure 3E,F). Studies have shown that activation of the liver bile acid synthesis pathway in NASH patients can result in increased reactive oxygen species (ROS) and stimulate fibroblasts, leading to liver fibrosis, a potential cause of end‐stage liver disease. Additionally, we found that although CYP7A1^+^ transitional cells from cluster 2 and cluster 5 both displayed activation of bile acid metabolism, the expression of FXR (NR1H4) was higher in CYP7A1^+^ transitional cells from cluster 2. In contrast, the expression of NR1H4 was lower in CYP7A1^+^ transitional cells from cluster 5 (Figure 3G). This suggests a failure of negative feedback regulation of bile acid metabolism in hepatocytes in precancerous lesions. Activation of FXR may serve as a potential therapeutic target for preventing NAFLD‐related hepatocellular carcinoma (HCC), rather than an effective treatment for patients in the early stages of NAFLD.

Consequently, we constructed a NAFLD disease progression model using HFD/DEN mice and collected liver tissues from mice fed with HFD for 8 weeks (NAFL), 24 weeks (NASH), and 32 weeks (HCC). Western blot detection revealed that the expression level of Cyp7a1 protein was higher in the liver of NASH mice compared to normal mice. Interestingly, the expression level of paracancer Cyp7a1 protein was higher but decreased in HCC tissues (Figure 3H).

In conclusion, hepatocytes undergo systematic metabolic changes during the transition from normal cells to malignant cells. The intricate equilibrium between fatty acid metabolism and bile acid metabolism is crucial in regulating the progression of NAFLD.

### 3.4 Progressive activation of HIF1A by hepatocytes during NAFLD‐related HCC progression

**FIGURE 4:**
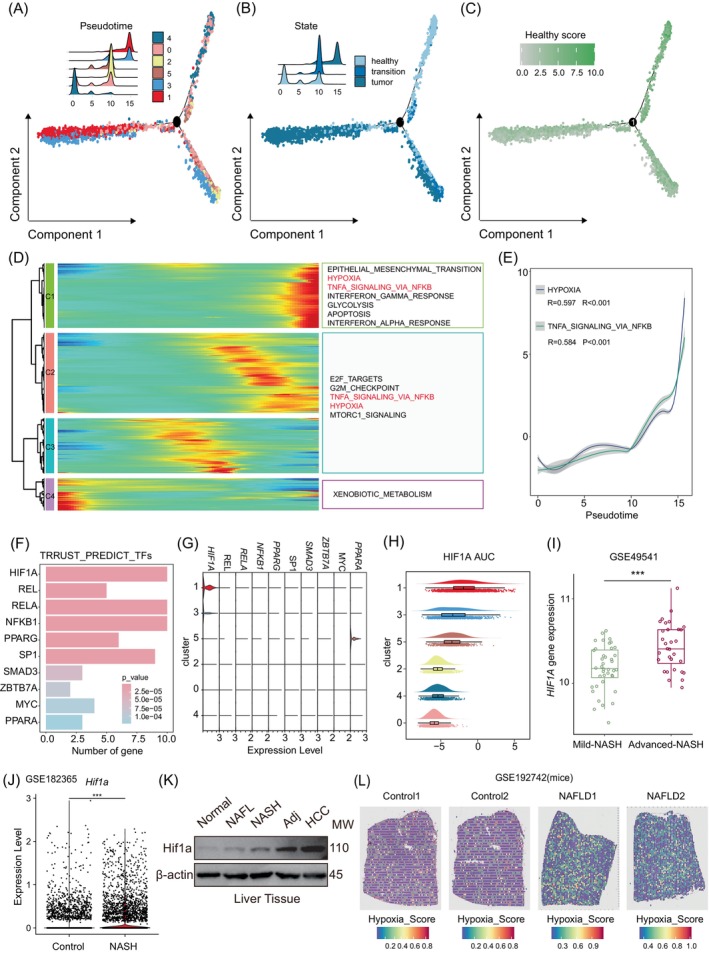
Hepatocytes upregulate HIF1A to adapt to the hypoxic microenvironment. (A) Pseudotime analysis of hepatocytes based on the Monocle 2 algorithm, and the density map showing the distribution and sorting of each cell cluster along the pseudotime trajectory. (B) Trajectory distribution of hepatocytes classification. (C) Trajectory distribution of Healthy_Score of hepatocytes. (D) The top 1000 differential genes whose expression levels vary most along the pseudotime trajectory were clustered into four clusters based on their expression trends, and the HALLMARK pathway activated by each gene in each cluster is shown on the right. (E) The degree of activation of HYPOXIA and TNFA_SIGNALLING_VIA_NFKB by Vision with the pseudotime change (F) Histogram showing the top 10 transcription factors affecting the activation of HYPOXIA and TNFA_SIGNALLING_VIA_NFKB pathways. (G) Violin plot showing the expression of key molecules in each cluster of hepatocytes. (H) Activation of transcriptional activity of HIF1A in hepatocytes as postulated by pySCENIC. (I) Box plot showing the expression of HIF1A in GSE49541 in NAFLD group and NASH group. (J) Violin plot shows Hif1a expression in the liver tissue of mice in the Control group and NASH group. (K) The expression of Hif1a protein in normal liver tissue, NAFL liver tissue, NASH liver tissue, NASH‐HCC‐Adj liver tissue and NASH‐HCC liver tissue were analysed by Western blot, and β‐actin was used as control. *N* = 3/group. (L) Hypoxia_Score in Vision fitting was mapped back to H&E images.

To gain deeper insights into the dynamics of hepatocytes and the mechanisms of tumorigenesis in the progression from NAFLD to HCC, we simulated the lineage differentiation process of hepatocytes using two independent pseudotime analysis methods. Both pseudotime analyses indicated that normal hepatocytes (cluster 1 and 4, identified as FABP1^+^ hepatocytes) were located at the initial segment of the differentiation trajectory, while transitional hepatocytes (cluster 2 and 5, identified as CYP7A1^+^ hepatocytes) were positioned in the middle segment. Malignant cells (clusters 1 and 3, identified as TFF3^+^ hepatocytes) were found at the terminal segment, suggesting that during NAFLD‐related HCC, hepatocytes undergo a transformation from their normal state to transitional intermediates, ultimately leading to malignancy. This progression aligns coherently with the categorization of hepatocellular entities as shown in Figure 2F,G. Notably, the gradual decline in the distribution of the healthy_score follows a discernible trend along the trajectory, thereby corroborating the accuracy of the findings from the pseudotime analysis (Figure 4A–C, Figure S4A–C). Subsequently, we performed an unsupervised clustering analysis of expression patterns of the top 1500 highly variable genes in hepatocytes. Furthermore, a functional enrichment analysis based on hallmark pathways was undertaken on each cluster. The intent was to uncover the dynamic alterations in the functional landscape of hepatocytes during the course of tumorigenesis (Figure 4D). Upon scrutinizing the gene expression profiles within hepatocytes, it was revealed that the C4 gene set, characterized by its purple designation, is notably elevated in expression within normal hepatocytes. Conversely, the C3 gene set (denoted by the blue designation) and the C2 gene set (denoted by the red designation) exhibit heightened expression levels in transitional cells. Finally, the C1 gene set (attributed to the green designation) displays marked upregulation within tumour cells. This nuanced distinction in gene expression patterns across different stages of NAFLD underscores the heterogeneity of hepatocellular responses. Gene set C4 exhibited elevated expression levels at the initial and manifested exclusive enrichment towards xenobiotic metabolic pathways related to liver metabolism, which was consistent with the definition of normal hepatocytes; the expression of C2 and C3 gene displayed augmentation in the middle stage of the pseudotime trajectory, and functional enrichment analysis show that this stage activated E2F targets, G2M checkpoint and TNFA Signalling via NFKB signalling pathways, and hepatocytes have undergone malignant transformation; gene set C1 showed high expression at the end of the pseudotime trajectory, which was mainly participated in pathways such as Epithelial mesenchymal transition, TNFA Signalling via NFKB, and Hypoxia‐associated oncogenic pathways (Figure 4D and Figure S4D). Notably, both the Hypoxia and TNFA Signalling via NFKB pathways were activated at the middle and end of the hepatocyte differentiation trajectory. Calculating the correlation between the hallmark pathway activation scores for Hypoxia and TNFA Signalling via NFKB pathways and the pseudotime revealed a significantly positive correlation (*R* > 0.5, *p* < 0.001) (Figure 4E). This observation suggests a progressive activation of the Hypoxia and TNFA Signalling via NFKB signalling pathways concomitant with the advancement of NAFLD. This underscores the conjecture that the pathological microenvironment marked by chronic inflammation and hypoxia assumes a pivotal role in the continuum of NAFLD‐related HCC progression, thereby potentially serving as a pivotal determinant in tumorigenesis. To substantiate this proposition, we conducted a comprehensive assessment of the correlation between the activation scores of all hallmark pathways and the pseudotime. The outcomes demonstrated a consistent and incremental activation of all hallmark pathways with the advancement of the disease (Figure S4F), indicating that the hallmark pathways intricately linked to tumorigenesis exhibit a gradual and synchronized activation throughout the course of disease progression.

Furthermore, we embarked on a more profound exploration into the conceivable mechanism underlying the influence of hypoxia on the malignant transition of intermediary cells. We predicted the pathway activation upstream transcription factors of HIF1A, RELA, NFKB1, and SP1 through the TRRUST database.^18^ The results showed that HIF1A, RELA, NFKB1, SP1 and other transcription factors may mediate Hypoxia and TNFA Signalling via NFKB signalling pathway in hepatocytes (Figure 4F and Figure S4G). Through a comprehensive assessment encompassing the significance of these transcription factors and their expression levels, we determined that HIF1A assumes a pivotal role solely in the malignant transformation of hypoxia‐regulated intermediary cells (Figure 4G). Subsequently, activation of transcription factor HIF1A in hepatocytes was predicted by pySCENIC.^29^ This analysis revealed heightened HIF1A activity within hepatocytes from clusters 5, 3, and 1 (Figure 4H). Interestingly, the HIF1A regulon activity demonstrated a progressive increase coinciding with the malignancy evolution of hepatocytes. This correlation exhibited a strong positive trend with disease progression, indicating a close linkage between HIF1A activation and hepatocellular malignant transformation.

Moreover, we validated the aforementioned conclusions using multiple independent external datasets. Firstly, we examined the expression of HIF1A in the livers of NAFLD patients in public datasets GSE48452,^14^ GSE66676,^15^ GSE89632,^13^ and GSE49541.^16^ The results consistently demonstrated a gradual increase in HIF1A expression with the progression of NAFLD‐related diseases in these patients (Figure 4I, Figure S4I–K). Furthermore, these findings were corroborated in a NASH mouse model exposed to a high‐sugar diet (HSD). Hif1a expression in hepatocytes of NASH mice exhibited a significant elevation compared to control mice (Figure 4J), aligning with the results in human samples. Additionally, using the NAFLD‐related HCC progression mouse model constructed based on the STAM mouse model,^44^ we observed a progressive increase in Hif1a expression during the disease progression of NAFLD, further confirming the upregulation of HIF1A expression in the development and advancement of NAFLD (Figure S4M). This cumulative evidence substantiates the crucial involvement of HIF1A in the progression of NAFLD. Additionally, the result was confirmed by western blot analysis (Figure 4K).

Subsequently, we calculated the hypoxia_score using the hypoxia signature proposed by Qian Zhang et al.^45^ The results demonstrated a significant positive correlation between the hypoxia_score and the malignant progression of hepatocytes (Figure S4H). Furthermore, by integrating spatial transcriptomic information from the livers of NASH mice, we confirmed the presence of hypoxia in the early stages of NAFLD, suggesting an already established hypoxic liver microenvironment (Figure 4L). In summary, as NAFLD advances, liver tissue hypoxia becomes more pronounced. Hepatocytes respond adaptively to the pathological environment of progressive hypoxia by upregulating the transcription factor HIF1A, thereby promoting the malignant transformation of hepatocytes into transitional and tumour cells. The pathological microenvironment characterized by liver hypoxia and the activation of the regulator HIF1A was closely associated with the development of NASH and HCC.

### 3.5 HIF1A reprograms the metabolism, proliferation and angiogenesis capacity of hepatocytes

**FIGURE 5:**
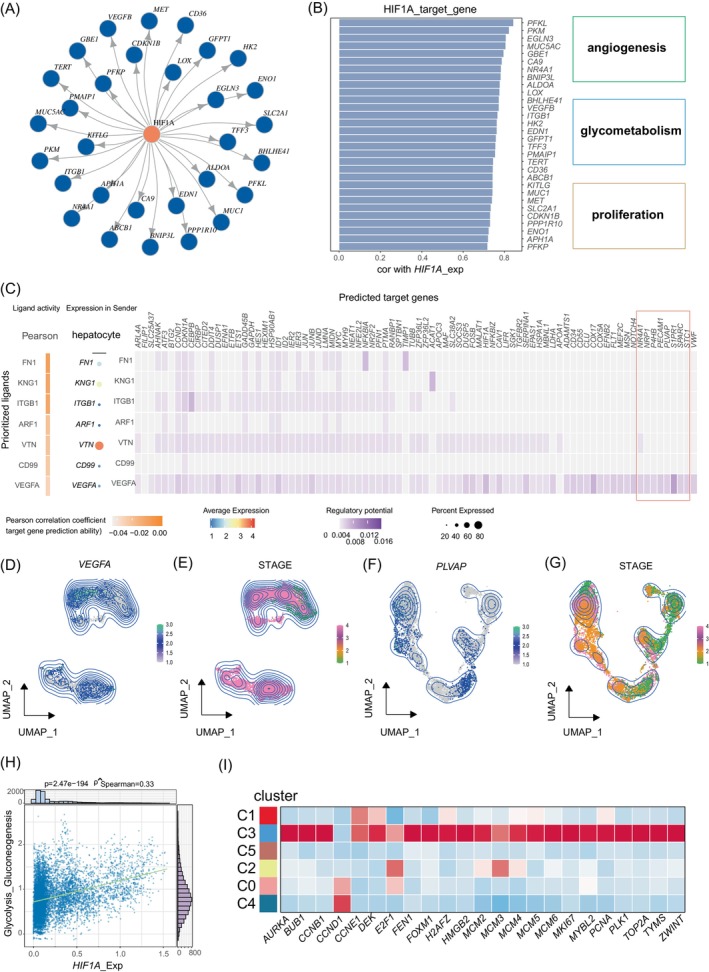
Hypoxia induces changes in the metabolism, proliferation and pro‐angiogenesis ability of hepatocytes. (A) Network diagram showing the downstream target genes activated by the transcription factor HIF1A. (B) Histogram showing the correlation of downstream target genes activated by the transcription factor HIF1A and activity of HIF1A (left). The right side is the gene‐related pathway (right). (C) NicheNet heatmap of the interaction between tumour cells and endothelial cells. (D) Density plot demonstrating the expression of *VEGFA* in hepatocytes. (E) Density plot demonstrating the disease stage distribution of hepatocytes. (F) Density plot demonstrating the expression of *PLVAP* in endothelial cells. (G) Density plot demonstrating the disease stage distribution of endothelial cells. (H) Scatterplot demonstrating the correlation between the AUC activity of *HIF1A* and the degree of glycolysis activation. (I) Heatmap showing the average expression of proliferation‐related genes in each cluster of hepatocytes.

Hypoxia is a crucial transcription factor that shapes the immune environment of the Tumour Microenvironment (TME) and can disrupt liver homeostasis.^46^ However, the specific impact of the hypoxic TME associated with NAFLD‐related HCC on tumour cells remains largely unexplored. In this study, we investigated the effects of activating the transcription factor HIF1A in hepatocytes on liver function. Firstly, we identified the genes highly correlated with HIF1A activity in hepatocytes and obtained a list of 30 target genes downstream of HIF1A by combining them with the transcriptional regulatory network of the Dorothea database^30^ (Figure 5A). These genes demonstrated a strong correlation with the activation of pathways involving cell proliferation, glucose metabolism, and pro‐angiogenesis (Figure 5B). This suggests that changes in HIF1A activation status have significant effects on hepatocyte metabolism, proliferation dynamics, and pro‐angiogenic potential, laying the foundation for tumorigenesis. We also measured the degree of activation of KEGG metabolic‐related pathways in hepatocytes. We found that metabolic pathways such as alanine, aspartic, and glutamate metabolism were significantly activated in normal hepatocytes but decreased in malignant cells. In contrast, pathways like glutathione metabolism and glycolysis exhibited activation within malignant cells (Figure S5A), indicating a close relationship between hepatocyte metabolic changes and tumorigenesis. Notably, the high expression of glycolytic key enzymes such as PFKL and GAPFH, downstream of HIF1A, was identified as the driving force behind the increased activation of glycolytic metabolic pathways within tumour cells (Figure 5H, Figure S5B,C). This elevated glycolytic activity provides the necessary energy to sustain the rapid proliferation and invasive potential of tumour cells. The expression of HIF1A was also strongly correlated with the proliferative capacity of hepatocytes, and genes related to proliferation were highly expressed in malignant cell clusters (Figure 5I). In addition, we conducted a NicheNet cell interaction analysis between tumour cells and endothelial cells^24^ to predict the cross‐talk between these two cell types using ligand and target gene expression information. The NicheNet prediction results revealed a strong interaction between hepatocytes and endothelial cells. Importantly, VEGFA, downstream of HIF1A, was identified as a crucial factor potentially triggering the activation of genes such as NR4A1, PECAM1, PLPP3, PLVAP, and STC1 within endothelial cells (Figure 5C), promoting angiogenesis in HCC. Furthermore, we observed increased expression levels of VEGFA and PLVAP with disease progression (Figure 5D–G), suggesting that continuous hypoxia induces increased secretion of VEGFA by tumour cells and activates the VEGFA‐PLVAP axis to promote angiogenesis. These findings suggested that transitional cells activate the Hedgehog pathway to promote angiogenesis.

In conclusion, the hypoxic microenvironment exerts a substantial influence on the transcriptome of hepatocytes. In response, hepatocytes adapt to hypoxia by activating the transcription factor HIF1A. The persistent activation of HIF1A subsequently affects the expression of specific gene networks, resulting in the activation of glycolytic metabolic pathways, increased proliferative capacity, and enhanced pro‐angiogenesis in hepatocytes.

### 3.6 Hypoxia‐induced immunosuppressive macrophages are enriched in tumour tissues

**FIGURE 6:**
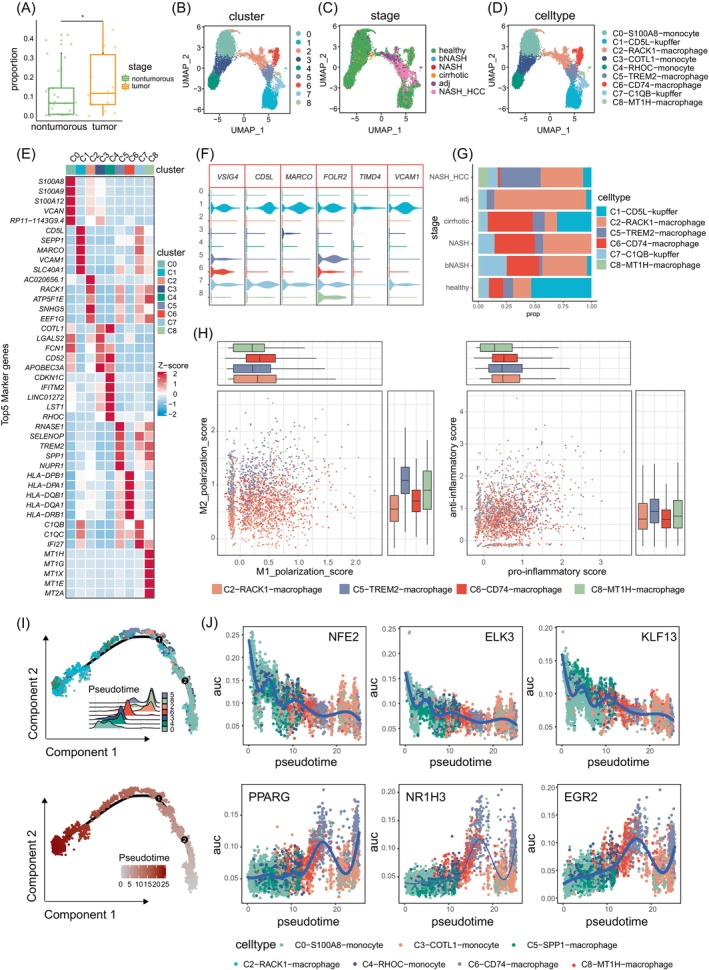
scRNA‐seq analysis of macrophages during the progression of NAFLD‐related HCC. (A) Boxplot depicting the proportions of liver macrophages in tumour and non‐tumour tissues. (B) UMAP plot showing the subdivision of macrophages into 9 clusters. (C) UMAP plot showing the distribution of macrophages at different disease stages in the context of NAFLD‐related HCC with different colours represent disease stages. (D) UMAP plot showing the subpopulation distribution of macrophages at different stages of NAFLD‐related HCC. (E) Heatmap showing the average expression of the top 5 differentially expressed genes in macrophages across various clusters, and the expression values are normalized. (F) Violin plot showing the expression profiles of characteristic genes for tissue‐resident macrophages. (G) Stacked bar chart showing the composition of macrophage subpopulations at different stages of NAFLD‐related HCC progression. (H) Scatter plot showing the pro‐/anti‐inflammatory scores and M1/M2 polarization scores of four monocyte‐derived macrophage subtypes. (I) Pseudotime analysis of hepatocytes based on the Monocle 2 algorithm on monocytes/macrophages, presenting the trajectory distribution of cell types (top) and the predicted pseudotime (bottom). (J) Transcription factors identified based on the SCENIC as significantly inhibited or activated during myeloid cell differentiation within the myeloid lineage process.

The macrophage population in the liver can be categorized into two main groups: liver tissue‐resident macrophages (known as Kupffer cells) and monocyte‐derived macrophages (MDM cells). Under normal conditions, Kupffer cells are the primary macrophage population in the liver. However, during the initiation of HCC, infiltrating monocytes/macrophages become the predominant stromal cells within the hepatocellular carcinoma (HCC) tumour microenvironment, and their presence is closely associated with the development and progression of HCC. As a result, these cells are considered significant potential targets for the treatment of hepatocellular carcinoma.^47^, ^48^, ^49^ To investigate the role of myeloid cells, including monocytes/macrophages, in the progression of NAFLD‐related HCC, we compared the cellular proportions of tumour‐infiltrating macrophages and liver macrophages in the non‐tumour state. We observed a specific increase in tumour‐infiltrating macrophages (Figure 6A). Subsequently, we re‐clustered myeloid cells and identified nine distinct subpopulations based on their highly expressed genes. These subpopulations were categorized as follows: C0‐S100A8‐monocyte, C1‐CD5L‐Kupffer, C2‐RACK1‐Mφ, C3‐COTL1‐monocyte, C4‐RHOC‐monocyte, C5‐TREM2‐Mφ, C6‐CD74‐Mφ, C7‐C1QB‐Kupffer, and C8‐MT1H‐Mφ. Among these subpopulations, clusters 0, 3, and 4 represented monocytes, with prominent expression of genes such as S100A8 and S100A9. Clusters 1 and 7 encompassed Kupffer cells, characterized by upregulated expression of genes like VSIG4 and CD5L. The remaining subpopulations were identified as monocyte‐derived macrophages (Figure 6B,D and E). We then examined the abundance of all macrophage populations, including monocyte‐derived macrophages and Kupffer cells, at different stages of the disease. The results showed specific enrichment of C5‐TREM2‐Mφ and C8‐MT1H‐Mφ in HCC. In contrast, C2‐RACK1‐Mφ and C6‐CD74‐Mφ were enriched in NASH and liver cirrhosis stages (Figure 6C,G, and Figure S6A), suggesting the presence of stage‐specific macrophage subpopulations that infiltrate the tissue at different stages of the disease. By evaluating the pro‐inflammatory and anti‐inflammatory scores, as well as the M1/M2 polarization scores of the macrophage subpopulations,^50^ we discovered that C2‐RACK1‐Mφ and C6‐CD74‐Mφ exhibited a more pro‐inflammatory phenotype and a skew towards M1 polarization (Figure 6H). In contrast, tumour tissue‐enriched C5‐TREM2‐Mφ and C8‐MT1H‐Mφ macrophages displayed a more anti‐inflammatory phenotype, with high expression of genes such as CD163, MARCO, and CSF1R, indicative of an M2‐like phenotype (Figure 6H). This suggests that the latter subpopulations have stronger tumour‐promoting effects compared to C2‐RACK1‐Mφ and C6‐CD74‐Mφ. This finding is consistent with previously reported immunosuppressive functions of SPP1^+^ TREM2^+^ macrophages in non‐small cell lung cancer and cholangiocarcinoma.^50^, ^51^

In order to investigate the polarization mechanism of monocyte‐derived macrophages, we isolated monocytes and monocyte‐derived macrophages for pseudotime analysis using monocle2.^27^ The results of the pseudotime analysis demonstrated the gradual differentiation of S100A8 ^+^ monocytes into TREM2 ^+^ macrophages with an M2 phenotype (Figure 6I). Moreover, by examining the key transcription factor motifs identified by pySCENIC, we observed the suppression of motifs such as NFE2, ELK3, and KLF13, alongside the persistent activation of motifs such as PPARG, NR1H3, and HIF1A, which may be responsible for M2 polarization (Figure 6J and Figure S6B). Importantly, previous research has established a significant association between these persistently activated motifs and hypoxia, implying a potential correlation between a hypoxic pathological microenvironment and macrophage M2 polarization. To further investigate this relationship, we calculated the hypoxia_score of macrophages. The results indicated that C5‐TREM2‐Mφ and C8‐MT1H‐Mφ enriched in tumour tissue had higher hypoxia_scores, suggesting that these cells were in a hypoxic tumour microenvironment. Furthermore, C5‐TREM2‐Mφ exhibited stronger antigen presentation and phagocytosis abilities (Figure S6C).

Finally, we investigated the potential interactions between macrophages and hepatocytes. By examining the ligand expression profiles of both cell types, we identified specific expression of inflammation‐related chemokines, including CXCL10, CCL4, and CCL5, within the fifth cluster, characterized by inflammatory transitional hepatocytes. This suggests that transitional hepatocytes could recruit C6‐CD74‐Mφ macrophages of the M1 phenotype through the CCL5/CCL16‐CCR1 axis (Figure S6D,F). Additionally, we observed a gradual increase in ligands such as CXCL1, CXCL3, CXCL5, CXCL16, CCL15, and CCL20 during the malignant transformation of hepatocytes. Among the receptors for these ligands, only CCR1 was specifically expressed in C5‐TREM2‐Mφ macrophages (Figure S6E,F). Consequently, this suggests that tumour cells may recruit suppressive macrophages through CCL15‐CCR1 in order to form an immunosuppressive microenvironment.

Subsequently, we corroborated this observation with microarray data from NASH‐associated mouse models. The expression of the chemokine CCL15 displayed a positive correlation with disease progression, and the deconvolution results revealed a highly positive correlation between the proportion of tumour cells and the proportion of TREM2^+^ macrophages (Figure S6G).

In conclusion, during the progression of NAFLD disease, macrophages exhibit distinct characteristics at different stages. Under normal physiological conditions, CD5L^+^ Kupffer cells play a role in maintaining liver homeostasis. In the NASH stage, RACK1^+^ macrophages with M1 phenotype become enriched in the liver, promoting liver inflammation. Upon the development of HCC, the hypoxic microenvironment triggers M2 polarization in macrophages, and tumour cells recruit TREM2^+^ macrophages with an M2 phenotype through the CCL15‐CCR1 axis, which promotes the formation of an immunosuppressive microenvironment and facilitates immune evasion by tumour cells. Blocking the CCL15‐CCR1 axis could inhibit the interaction between tumour cells and macrophages, suppressing the formation of a tumour immunosuppressive microenvironment, and impeding the growth and progression of the tumour. Therefore, targeting the CCL15‐CCR1 axis may hold therapeutic potential for NAFLD‐related HCC.

## 4 DISCUSSION

Nonalcoholic fatty liver disease (NAFLD), a prevalent chronic liver ailment, is a growing concern in global healthcare.^52^, ^53^, ^54^ NAFLD encompasses simple fatty liver, nonalcoholic steatohepatitis (NASH), and its associated cirrhosis, all of which can eventually lead to the development of hepatocellular carcinoma (HCC).^55^, ^56^ Compared to HCC originating from other causes, NAFLD‐related HCC has lower survival rates and a reduced likelihood of liver transplantation.^57^ Furthermore, recent clinical trials have demonstrated that immune checkpoint inhibitor therapy is not suitable for NAFLD‐related HCC.^9^ Importantly, there are currently no approved drugs for the treatment of NASH.^58^ Therefore, understanding the pathogenesis of NAFLD‐related HCC is crucial in the pursuit of targeted therapeutic interventions.

In this study, we collected and integrated single‐cell RNA sequencing (scRNA‐seq) data from a total of 31 liver samples. The sample distribution consisted of 14 samples from healthy individuals, 3 samples from bNASH patients, 3 samples from NASH patients, 2 samples from NAFLD‐related cirrhosis patients, and 9 samples from NAFLD‐related HCC patients. By utilizing scRNA‐seq data obtained from these liver samples at various stages of NAFLD progression, we constructed a comprehensive single‐cell transcriptome map that delineates the progression of NAFLD‐related HCC. Our primary objective was to identify the key cell subsets closely associated with the disease progression. Hepatocytes, which account for approximately 80% of the liver's volume and 60% of its cell composition, play a critical role in maintaining liver homeostasis.^37^, ^59^ However, the existing single‐cell liver disease data sets often fail to adequately represent hepatocytes since many studies based on single‐cell sequencing adopt a sorting strategy prior to sequencing, thereby mitigating the impact of hepatocytes on the sequencing results.^9^, ^32^, ^60^, ^61^ We aimed to address this limitation by systematically analysing the transcriptional heterogeneity and possible dynamic transformation of hepatocytes during the progression of NAFLD. Our analysis revealed that hepatocytes exhibit continuous dynamic changes throughout the occurrence and development of NAFLD, distinguishing them from other cells that display phasic characteristics unique to NAFLD. Notably, CYP7A1^+^ hepatocytes were found to be in a transitional state of precancerous lesions, exhibiting characteristics of tumour cells and inflammation. These findings suggest that this particular subset of hepatocytes may play a crucial role in driving the progression of the disease. However, it is important to note that further verification of these conclusions is necessary through additional data analysis and external experiments.

In recent years, extensive research has demonstrated that bile acids, in addition to their normal physiological functions, are closely associated with the development of liver diseases.^62^, ^63^ Patients with impaired liver function exhibit significantly different metabolic components of bile acids compared to healthy individuals, and the abnormal increase in specific bile acid components has been strongly implicated in the onset of HCC.^64^ This study made a noteworthy observation that the primary bile acid synthesis pathway is specifically activated by CYP7A1^+^ hepatocytes, while the expression of FXR decreases as the bile acid synthesis pathway progresses into precancerous lesions. This reduction signifies the failure of the negative feedback regulatory mechanism governing the bile acid synthesis pathway, which plays a role in the inflammatory and carcinogenic transformation of NAFLD. Western blot (WB) experiments revealed that the expression level of CYP7A1 starts increasing at the stage of NASH, continues to rise until the onset of tumours, and subsequently declines during the tumour stage. This suggests that quantitative indicators based on the expression level of CYP7A1 and primary bile acid levels may serve as effective biomarkers for predicting HCC and diagnosing NASH at an early stage. Activation of FXR to inhibit bile acid synthesis in hepatocytes may potentially serve as a therapeutic target for preventing the malignant transformation of transitional hepatocytes. Further data analysis and experiments are necessary to explore the characteristics of transitional hepatocytes, with the aim of guiding the prevention, diagnosis, and treatment of NAFLD‐related HCC patients.

Our study also revealed that during the progression of NAFLD‐related HCC, hepatocytes adapt to the gradually hypoxic pathological microenvironment by upregulated HIF1A. HIF1A is a key transcription factor that controls hypoxia‐induced gene expression and triggers metabolic events critical for disease progression.^65^, ^66^ Hypoxia could lead to liver cell injury and glycolysis activation, which can increase the production of vascular endothelial growth factor, stimulate angiogenesis, and promote the growth, invasion, and metastasis of tumour cells, while glycolysis activation provides energy for this process.^67^ The persistent activation of HIF1A maintains a state of continuous transformation in the metabolic profile, proliferation capacity, and angiogenic potential of hepatocytes. This cumulative effect ultimately culminates in the development of advanced‐stage liver cancer.

The occurrence of chronic liver disease not only involves the lesion of hepatocytes but also is accompanied by the remodelling of other non‐parenchymal cells. The cellular interaction between hepatocytes and other non‐parenchymal cells maintains the balance of the physiological microenvironment or breaks the balance in the pathological environment.^68^ In particular, the pathology of NAFLD originates from the accumulation of toxic lipids in hepatocytes, which release signals to trigger pro‐inflammatory responses. Kupffer cells recognize liver injury and promote the recruitment of peripheral immune cells.^69^ Data from this study showed that macrophages in the normal liver were mainly the kupffer cells, which maintained liver homeostasis. C2‐RACK1‐Mφ, C6‐CD74‐Mφ of proinflammatory phenotypes were enriched in NASH, cirrhosis, and stages, which aggravated liver inflammation. Anti‐inflammatory phenotypes of C5‐TREM2‐Mφ and C8‐MT1H‐Mφ were specifically enriched in HCC, promoting immune escape and disease progression of hepatocellular carcinoma. The scRNA‐seq map of NASH‐related HCC constructed in this study provides insight into the dynamic changes of different cell types and signal crosstalk between cells at different periods. The data from this study suggest that malignant cells can recruit macrophages with proinflammatory phenotype via the CCL5/CCL16‐CCR1 axis under inflammation. Tumour cells can recruit inhibitory macrophages through CCL15‐CCR1. In addition, in the disease progression of NAFLD‐related HCC, the continuous hypoxia microenvironment induces the secretion of VEGFA by hepatocytes and activates the VEGFA‐PlVAP axis to promote angiogenesis, leading to impaired liver self‐repair function, ultimately promoting the progression of NAFLD‐related diseases.

In this study, we integrated single‐cell transcriptome sequencing data from the liver at various stages of NAFLD development, and constructed a single‐cell transcriptome atlas specifically focused on NASH‐related HCC development. The primary objective was to investigate the dynamic changes in cell components that occur as the liver progresses from a normal state to NAFLD‐related HCC. We aimed to gain a better understanding of the underlying development mechanism of NAFLD‐related HCC. This research provides a theoretical foundation and experimental basis for identifying biological targets, conducting drug research, and facilitating the clinical application of NAFLD‐related HCC.

## 5 CONCLUSION

This study collected and integrated liver single‐cell transcriptome sequencing data at different stages of NAFLD‐related HCC progression to construct a single‐cell transcriptome atlas of NAFLD‐related HCC disease progression, revealing a transitional CYP7A1 ^+^ hepatocytes in the precancerous state. These cells exhibited characteristics shared by hepatocytes in both NASH and HCC stages, marked by heightened CYP7A1 expression and upregulated primary bile acid synthesis pathways. Activation of FXR inhibits bile acid synthesis in hepatocytes, which might be a potential therapeutic target to prevent the malignant transformation of transition cells. In addition, the liver pathological microenvironment becomes progressively hypoxic during the progression of NAFLD‐related HCC, and hepatocytes adapt to the progressive hypoxia pathological microenvironment by upregulating HIF1A, which leads to changes in the metabolic level, proliferation potential, and pro‐angiogenic ability of hepatocytes, induced end‐stage liver disease. Tumour cells regulate endothelial cells through the HIF1A‐VEGFA‐PLVAP axis, instigating angiogenesis and fueling tumour growth. At the same time, hypoxia can lead to M2 polarization of macrophages and recruitment by tumour cells depending on the CCL15‐CCR1 axis, promoting the formation of a tumour immunosuppressive microenvironment. This study deeply analysed the dynamic changes of hepatocytes and macrophages during the progression of NAFLD‐related HCC and highlighted the potential regulatory mechanism of the hypoxic disease microenvironment and the activation of transcription factor HIF1A on the occurrence and development of NAFLD‐related HCC. These insights provide a robust theoretical foundation and empirical basis, essential for the development of novel drugs and potential clinical applications for addressing NAFLD‐related HCC.

## AUTHOR CONTRIBUTIONS

Ling Lu, Haoming Zhou, Yuan Liang and Rui Zhang conceived and designed the study. Siddhartha Biswas, Qingfa Bu and Zibo Xu carried out all experiments and wrote the manuscript. Yuan Liang, Rui Zhang, Lei Qiao and Jiaqi Tang participated in bioinformatics analysis. Yan Zhou and Jinren Zhou supported the study. Qingfa Bu, Zibo Xu, Yan Zhou and Jinren Zhou revised the manuscript. All authors read and approved the final manuscript.

## FUNDING INFORMATION

This study was supported by grants from the National Natural Science Foundation of China (81971495), the CAMS Innovation Fund for Medical Sciences (2019‐I2M‐5‐035), the State Key Laboratory of Reproductive Medicine (SKLRM‐K202001), the Foundation of Jiangsu Collaborative Innovation Center of Biomedical Functional Materials, and 2022 Jiangsu Graduate Research and Innovation Program (SJCX22_0668, KYCX22_1842).

## CONFLICT OF INTEREST STATEMENT

The authors declare that they have no competing interests.

## Supporting information


**Data S1.** Supporting Information

**Table S1.** Data resource.
**Table S2.** Cell type markers.
**Table S3.** Signature genes.

## Data Availability

The code used in our study can be obtained from the corresponding author. The data sets used in the present research were summarized in Table S1.
