# Supplementary material for: Integrated single‐cell transcriptomics reveals the hypoxia‐induced inflammation‐cancer transformation in NASH‐derived hepatocellular carcinoma
Source: Cell Prolif. 2023 Nov 22;57(4):e13576. doi: 10.1111/cpr.13576 (PMC10984103; doi:10.1111/cpr.13576)
Supplement: Supplementary file 1 — Data S1. Supporting Information [file CPR-57-e13576-s001.pdf]

SUPPLEMENTAL DATA

Integrated Single-cell Transcriptomics Reveals the Hypoxia-induced Inflammation-Cancer Transformation in NASH-Derived Hepatocellular Carcinoma

Yuan Liang, Rui Zhang, Siddhartha Biswas, Qingfa Bu, Zibo Xu, Lei Qiao, Yan Zhou, Jiaqi Tang, Jinren Zhou, Haoming Zhou, Ling Lu

Corresponding authors: **Ling Lu**, MD, PH. D. Affiliated Hospital of Xuzhou Medical University, Xuzhou, China, and Hepatobiliary Center, The First Affiliated Hospital of Nanjing Medical University, No. 300 Guangzhou Road, Nanjing 210029, China, **Email: lvling@njmu.edu.cn**.

The PDF file includes:

Supplemental Figures S1 to S6;

Supplemental Tables S1 to S3 (use excel sheets to submit the supplemental tables).

Supplemental Information

1. **Supplemental figures:**

Supplementary Figure1 The subsets of immune and non-immune cells.

Supplementary Figure2 Transcriptional characteristics of transition cells.

Supplementary Figure3 *CYP7A1* was highly expressed in transition cells.

Supplementary Figure 4 HIF1α is gradually activated in NAFLD-related diseases.

Supplementary Figure5 HIF1α reprograms hepatocytes.

Supplementary Figure6 Potential cell interactions between hepatocytes and macrophages.

2. Supplemental tables:

Supplemental table1：Data Resource

Supplemental table2：Cell Type Markers

Supplemental table3：Signature Genes

Supplemental Figures

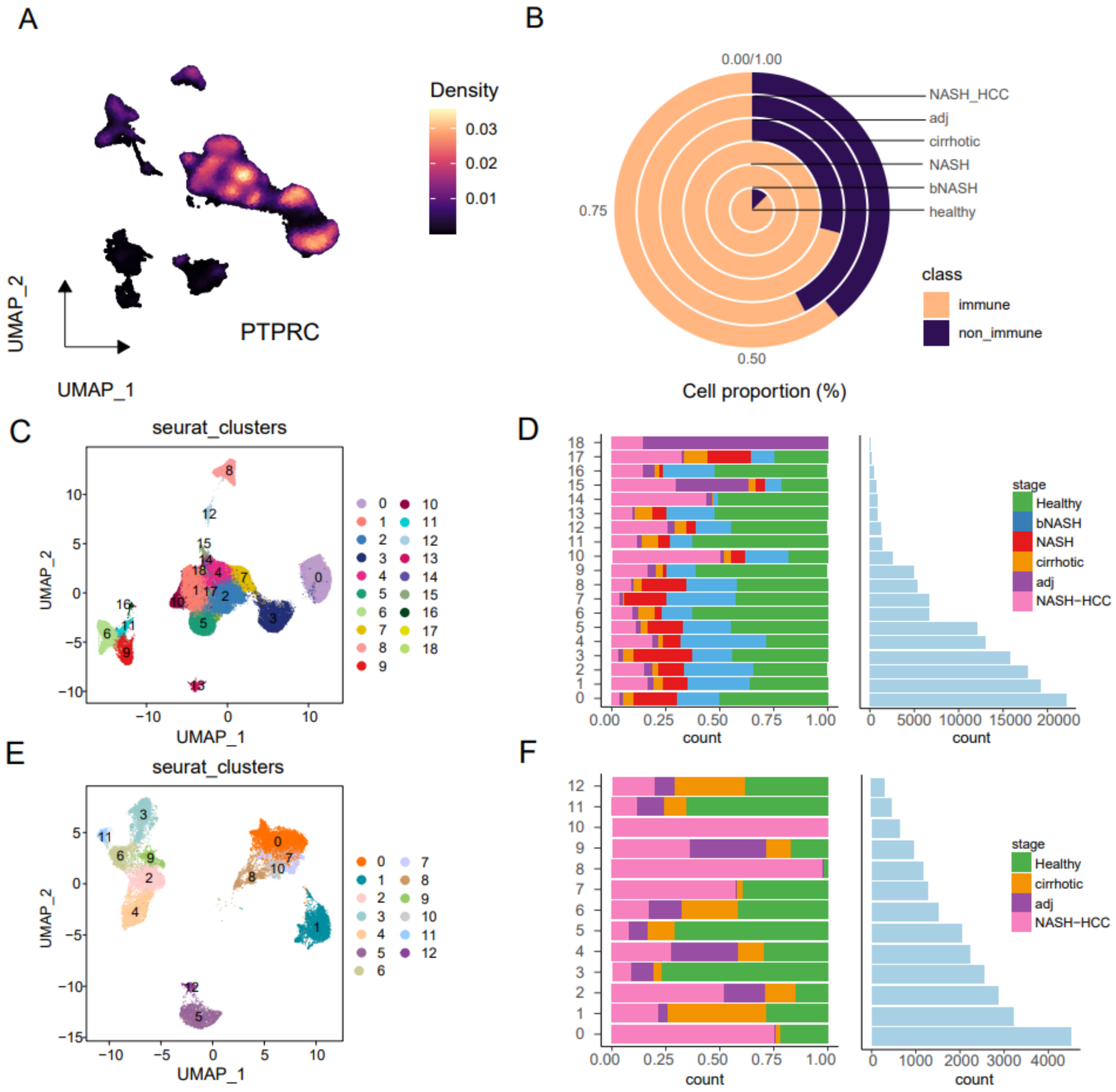

Supplementary Figure1 The subsets of immune and non-immune cells. (A) Density plot showing the distribution of *PTPRC*. (B) The pie chart shows the proportion of immune cells in different stage samples. (C) UMAP plot of clustering immune cells at different stages of NAFLD-related HCC. (D) The stage composition of each cluster of immune cells (left), and the number of cells in each cluster of immune cells (right). (E) UMAP plot of clustering non-immune cells at different stages of NAFLD-related HCC. (F) The stage composition of each cluster of non-immune cells (left), and the

number of cells in each cluster of non-immune cells (right).

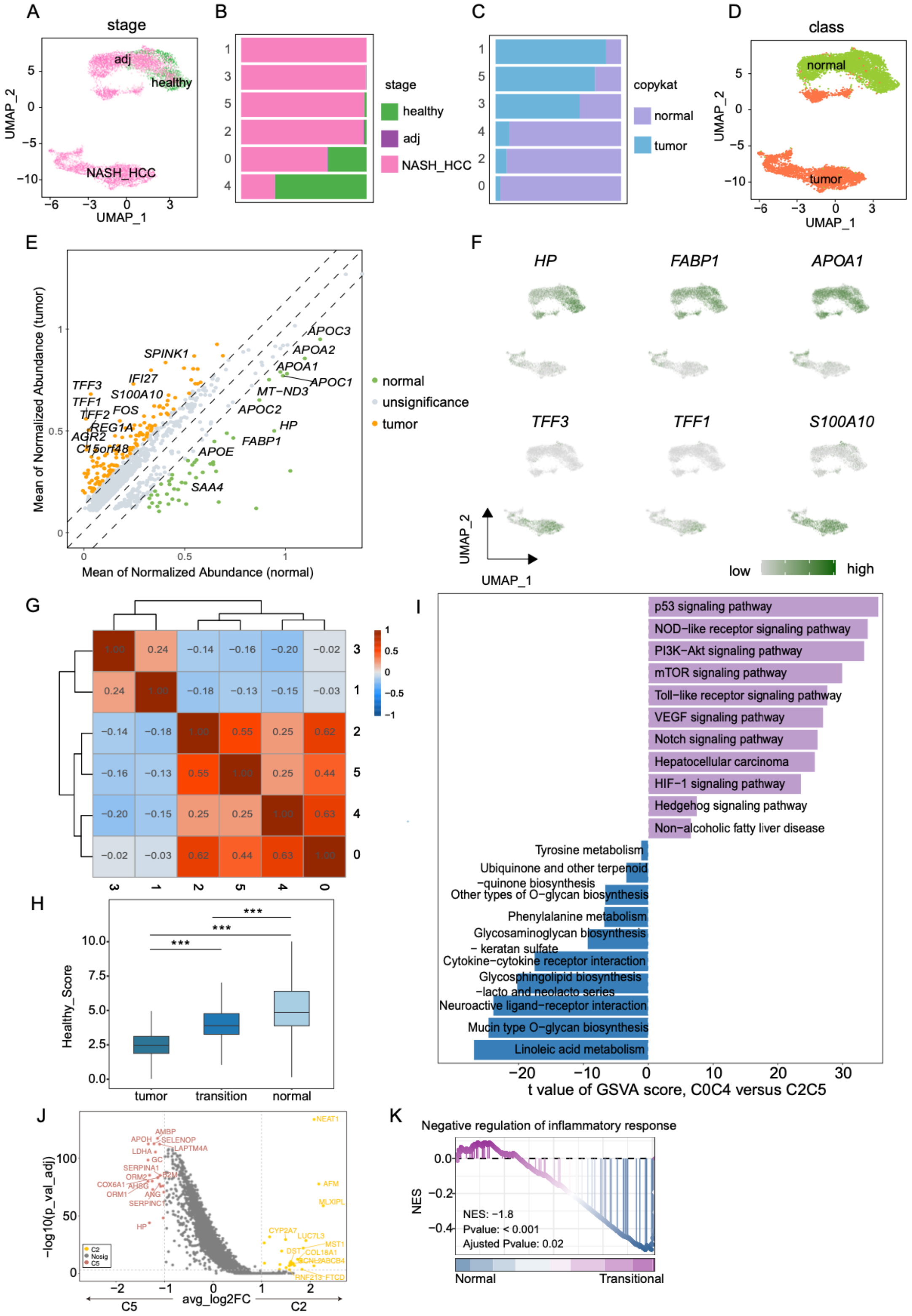

Supplementary Figure2 Transcriptional characteristics of transition cells. (A) UMAP plot showing the stage distribution of hepatocytes. (B) Bar graph showing the

stage composition of each cluster of hepatocytes. (C) Bar graph showing the chromosomal variation of each cluster of hepatocytes (D) UMAP plot showing the distribution of malignant and non-malignant cells in hepatocytes. (E) Scatterplot showing differential genes in malignant and non-malignant cells. (F) UMAP plot showing the expression distribution of differential genes of malignant and non-malignant cells. (G) Correlation heatmap showing the correlations between clusters of hepatocytes. (H) Boxplot showing the Healthy_Score in each subpopulation of hepatocytes. (I) Bar graph showing the differentially activated pathways of clusters C0 and C4 of hepatocytes and clusters C2 and C5 of hepatocytes. (J) Scatterplot showing differential genes in C2 and C5 hepatocytes. (K) GSEA analysis of the enrichment of Negative Regulation of Inflammatory Response gene sets between transitional hepatocytes and normal cells.

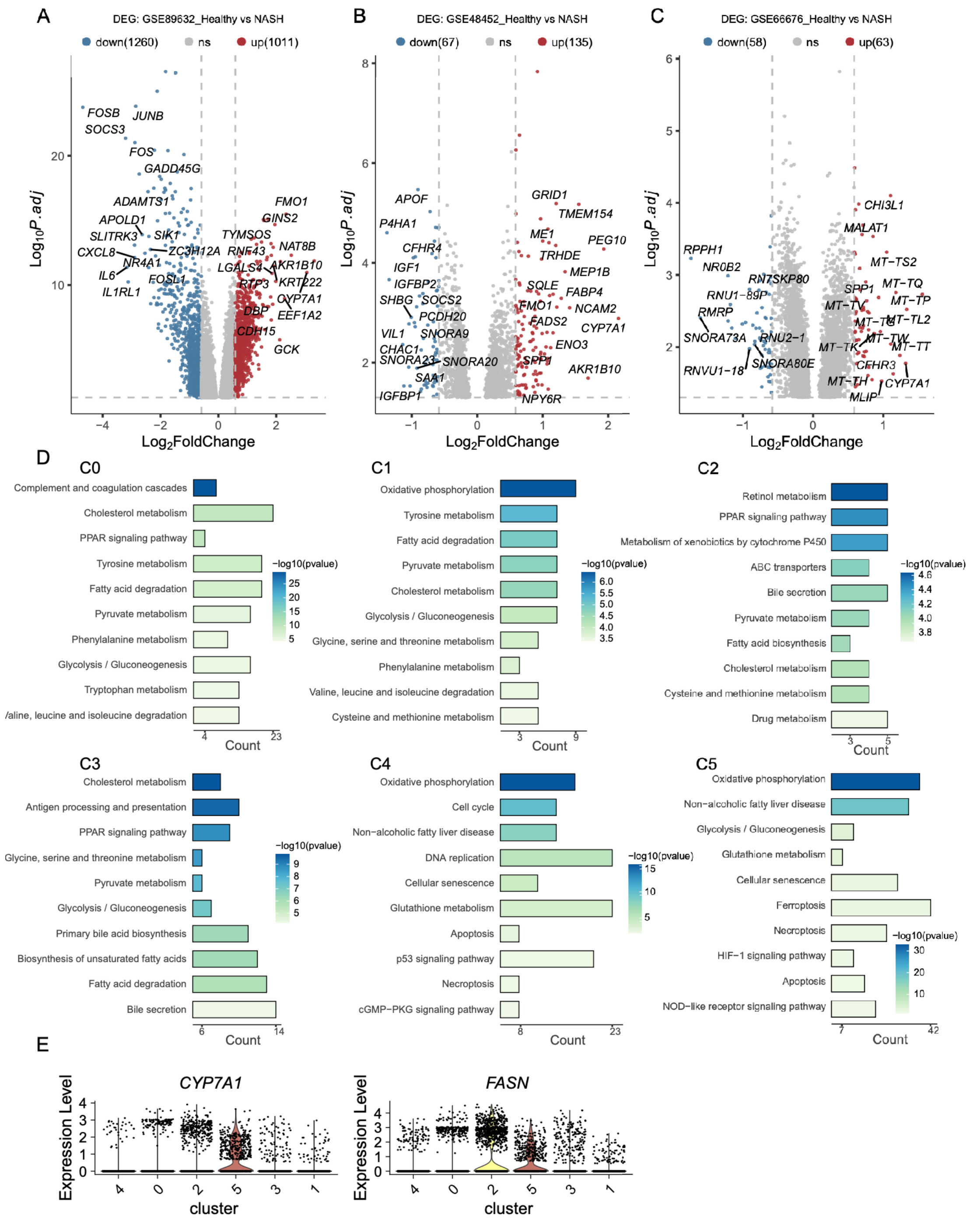

Supplementary Figure3 *CYP7A1* was highly expressed in transition cells. (A)Volcano plot shows the differential genes between NASH samples and Normal samples in the GSE48452 dataset. (B) Volcano plot shows the differential genes between NASH samples and Normal samples in the GSE66676 datasets. (C) Volcano plot shows the differential genes between NASH samples and Normal samples in the

GSE89632 datasets. (D) Bar graph showing the results of functional enrichment analysis of each cluster of hepatocytes. (E) Violin plot showed the expression of *CYP7A1* and FASN in hepatocytes.

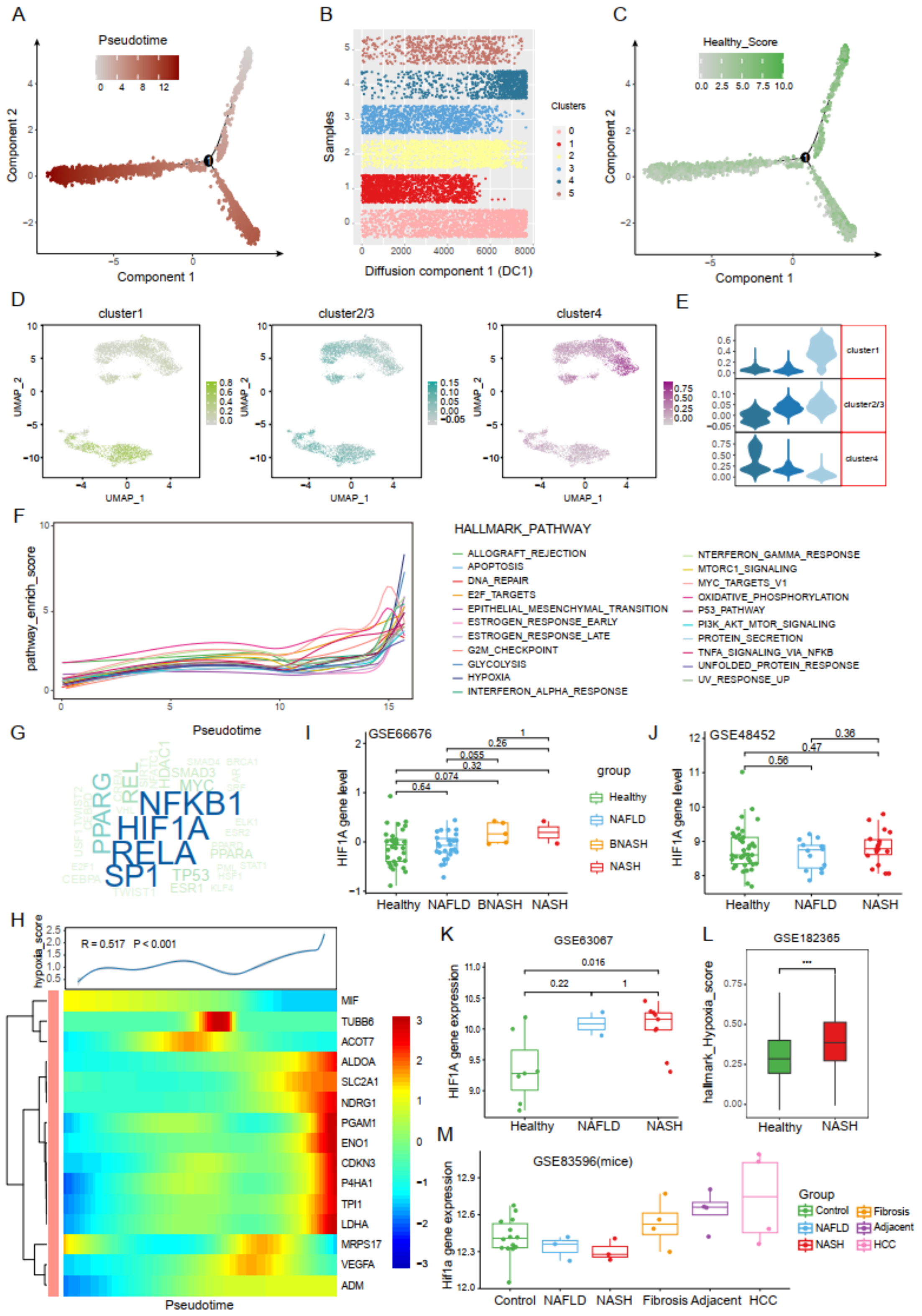

Supplementary Figure 4 HIF1α is gradually activated in NAFLD-related diseases.

(A) Prediction of pseudotime of hepatocytes based on Monocle2 algorithm. (B) Pseudotime ordering of hepatocytes predicted by the destiny algorithm. (C) Pseudotime analysis of hepatocytes based on the destiny algorithm. (D) UMAP plot showing the gene expression of each module in pseudotime analysis. (E) Violin plot showing the degree of gene module activation in different hepatocyte subpopulations. (F) Correlation of HALLMARK pathway activation with Pseudotime. (G) Key gene sets. (H) Heatmap showing correlations of hypoxia-related genes with Pseudotime. (I) Box plot showing the expression of *HIF1A* in Healthy group, NAFLD group, BNASH group and NASH group of GSE66676 datasets. (J) Box plot showing the expression of *HIF1A* in Healthy group, NAFLD group and NASH group of GSE48452 datasets. (K) Boxplot showed the expression of *HIF1A* in healthy group, NAFLD group and NASH group in GSE63067. (L) Boxplot showing the activation of hypoxia pathways in hepatocytes of Control and NASH mice in the GSE182365 dataset. (M) Boxplot showed the expression of *Hif1a* in GSE83596 in control group, NAFLD group and NASH group, cirrhosis group, adjacent group and tumor group.

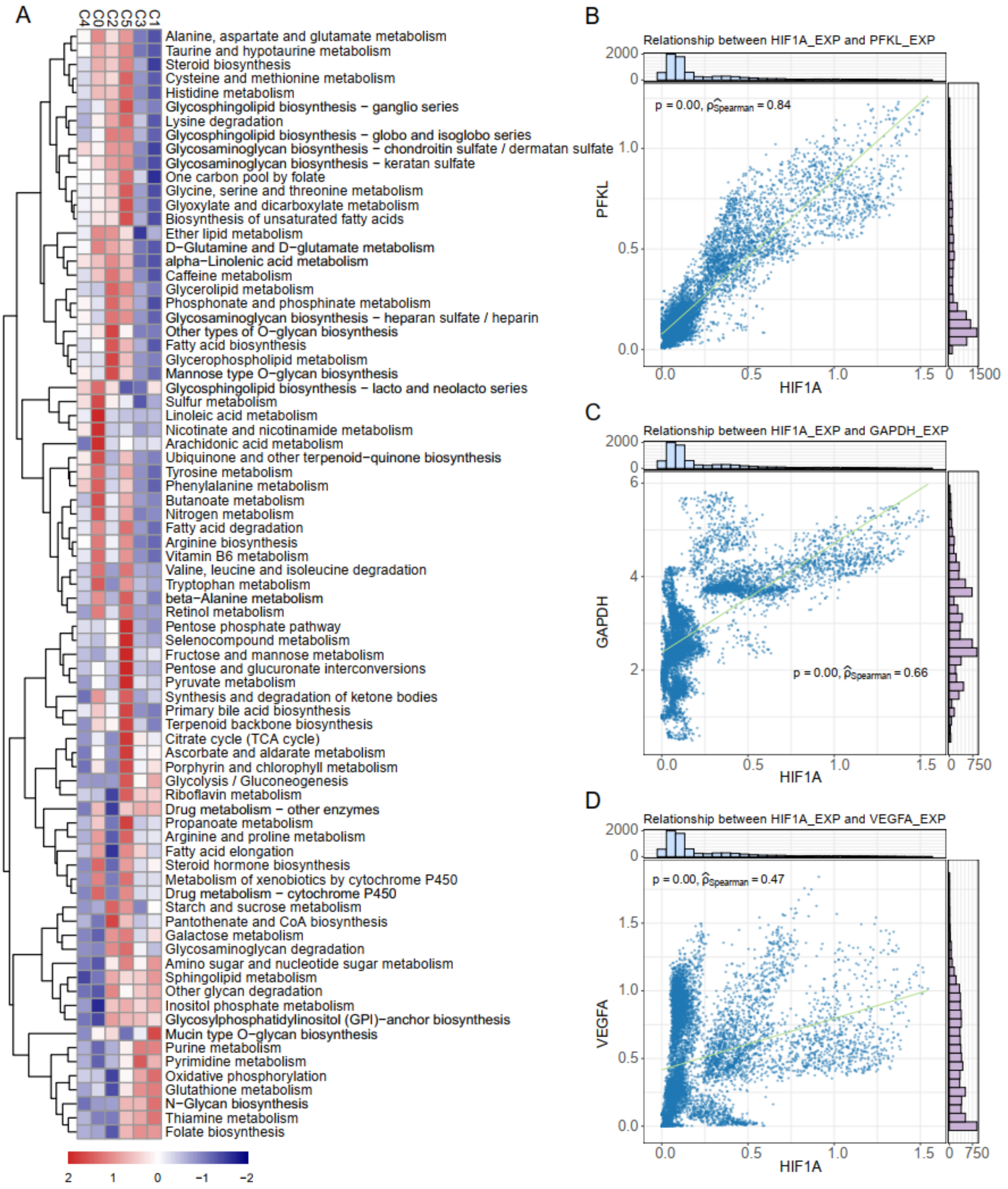

Supplementary Figure5 HIF1α reprograms hepatocytes. (A) Heatmap showing the activation of metabolic-related pathways in each cluster of hepatocytes in the KEGG database. (B-D) The correlation between the expression value of *HIF1A* and the expression value of *PFKL*, *GAPDH* and *VEGFA*.

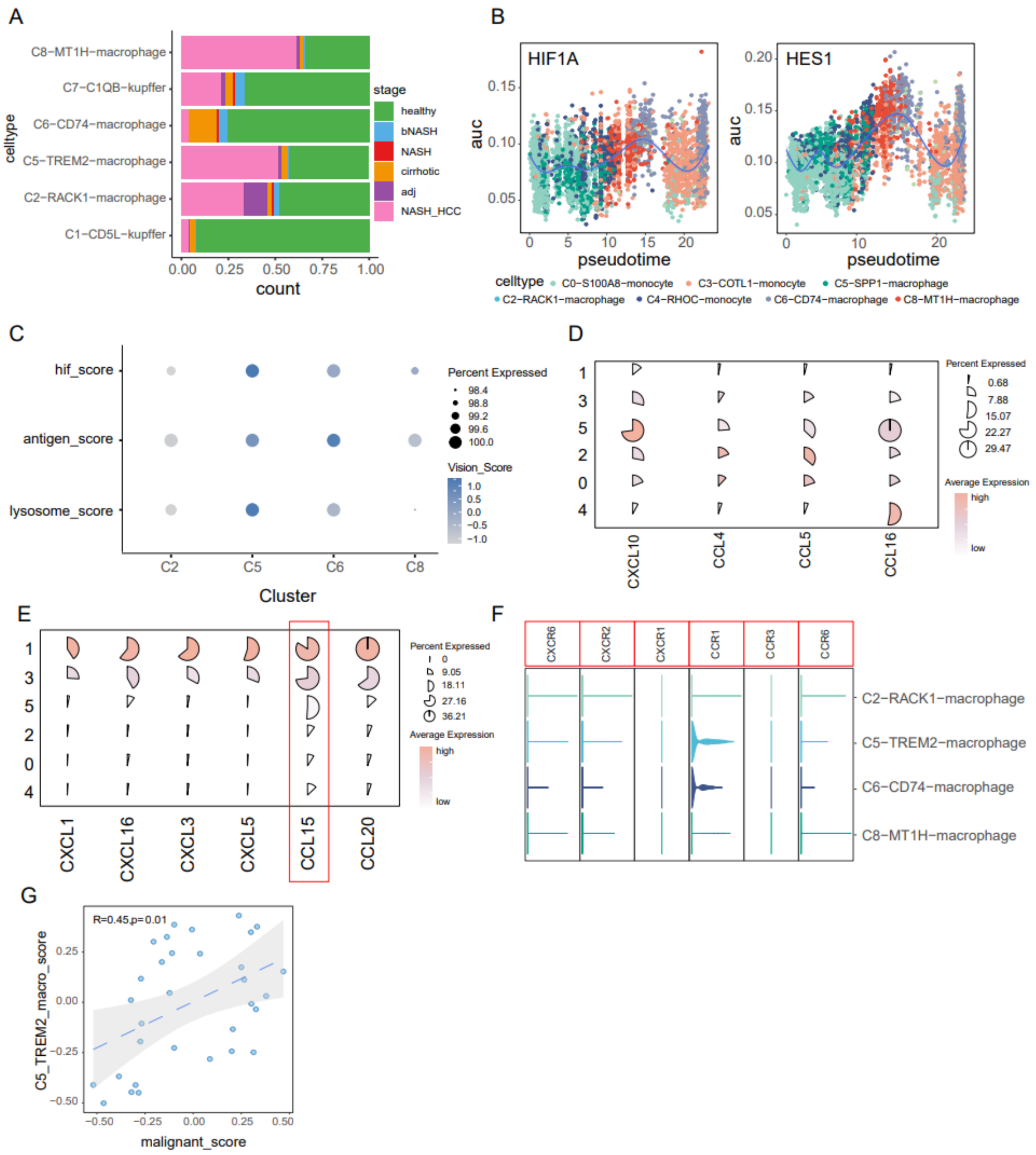

Supplementary Figure6 Potential cell interactions between hepatocytes and macrophages. (A) Stacked histogram showing the composition of macrophages at each stage of NASH-associated HCC progression. (B) Identification activated transcription factors during myeloid cell differentiation based on SCENIC. (C) Bubble plot showing hypoxia score, antigen presentation score, and phagocytosis score in macrophages. (D-E) Bubble diagrams show the expression of chemokines in each cluster of hepatocytes, where the size of the pie chart represents the percentage of

marker gene expression; the shade of color represents the average expression value of marker gene expression. (F) Violin plots showing the expression of corresponding ligands in macrophages. (G) Scatterplot showing the correlation of malignant cells and C5-TREM2-Mφ cells.
